# Phytolith Assemblages as a Promising Tool for Quantitative Canopy Coverage Reconstruction in Subtropical Forests, China

**DOI:** 10.3389/fpls.2022.912627

**Published:** 2022-06-20

**Authors:** Nannan Li, Fengling Yu, Dorothy Sack, Zhaoquan Huang, Ganghua Tian, Shengtao Liu

**Affiliations:** ^1^State Key Laboratory of Marine Environmental Science, College of Ocean and Earth Sciences, Xiamen University, Xiamen, China; ^2^Department of Geography, Ohio University, Athens, OH, United States

**Keywords:** phytoliths, tree coverage, quantitative reconstruction, subtropics, vegetation

## Abstract

This study investigates the reliability of phytolith assemblage analysis for characterizing subtropical vegetation and explores the potential for using these modern phytolith–vegetation relationships for paleoenvironmental interpretation in southeastern China. The samples were collected from five common subtropical vegetation communities in the Daiyun Mountains, southeastern China, with the above-ground vegetation recorded at each plot. Constrained ordination analysis was used to determine the most important factor governing the variations in phytolith assemblages that could be quantitatively reconstructed with weighted averaging partial least squares regression (WAPLS). The relationship between modern phytolith assemblages and the parent vegetation, as well as production, dispersal, and taphonomic processes, was discussed. Results demonstrated that the main subtropical biomes in southeastern China could be well distinguished by soil phytolith assemblages. In particular, the overall amount of tree coverage was well represented by topsoil phytolith assemblages. Grass silica short cell phytoliths (GSSCP) tended to occur in higher proportions in open habitats (shrub-meadow) at higher elevations, whereas non-grass phytolith morphotypes attained higher frequencies under mixed and broadleaf forests at lower elevations. Human-induced deforestation might increase the frequency of GSSCP within the bulk phytolith assemblage. Our results constitute the primary phytolith reference data for the subtropical zone in southeastern Asia where vegetation change during the Holocene period, particularly forest shifts, anthropogenic deforestation, and early agriculture are poorly documented.

## Introduction

Phytoliths are microscopic silica accumulations produced in many plants by the uptake of soluble monosilicic acid through roots and then precipitated as amorphous opal-A within or between plant cells and tissues (Piperno, 1988). Phytoliths occur in many plants but are especially abundant and diagnostic within Poaceae species (e.g., Twiss et al., 1969; Twiss, 1992; Barboni et al., 1999). As a result, fossil phytolith analysis has allowed researchers to distinguish between forest and grassland landscapes, C3 and C4 grasslands, and environments dominated by different subfamilies of Poaceae (e.g., Alexandre et al., 1997; Barboni et al., 1999; Strömberg, 2004; Boyd, 2005; Bremond et al., 2008; Dunn et al., 2015). In addition, because phytoliths remain well preserved in oxidizing conditions and soil aggregates (e.g., Li et al., 2020, 2022) and are diagnostic to the subfamily level (Twiss et al., 1969; Twiss, 1992; Barboni et al., 1999), they are increasingly recognized as a powerful tool for paleovegetation reconstructions that involve grasses (e.g., Alexandre et al., 1997; Barboni et al., 1999; Strömberg, 2004; Boyd, 2005; Bremond et al., 2008; Dunn et al., 2015; Li N. et al., 2018, 2021), especially for areas in which conventional pollen analysis is not an option.

Despite the valuable role of phytoliths in paleoecological reconstructions in grass-dominated ecosystems, whether phytoliths can aid in forest community differentiation has been questioned. A critical concern is that phytoliths may fail to reconstruct forest vegetation reliably where Poaceae species are absent or uncommon, as in shrublands (Bremond et al., 2004). Globally, the capability of phytolith assemblages to characterize a change in forest communities has been investigated but most of those studies have been conducted in the tropics (Bremond et al., 2004; Barboni et al., 2007; Crifò and Strömberg, 2021), especially in tropical Africa (e.g., Alexandre et al., 1997; Bremond et al., 2004, 2005; Barboni et al., 2007; Chen and Smith, 2013). Emphasis on the tropics may be attributed to the fact that phytoliths produced by tropical woody Palmaceae are easily differentiated using phytoliths. For extratropical vegetation, however, Palmaceae represent at most only a small portion of the vegetation community. Extratropical trees produce only a small amount of diagnostic phytoliths (e.g., Wang and Lu, 1993; Lu et al., 2006), and arboreal species from subtropical and temperate zones produce similar diagnostic types (e.g., Wang and Lu, 1993; Lu et al., 2006; Gao et al., 2018; Wen et al., 2018). Consequently, in extratropical forests where open-habitat grasses are absent or uncommon, very few topsoil-based phytolith–vegetation relationship calibrations are available (e.g., An et al., 2015; Dunn et al., 2015; Biswas et al., 2021; Pei et al., 2021), underscoring that extratropical region-specific calibration is still of great importance for future global dataset comparisons.

In East Asia, the broadleaved evergreen forest represents the climax vegetation of the subtropics and is a product of the monsoonal climate (Hou, 1982). Subtropical forest occupies 25% of China’s total land area and 71% of the total forest area in the global subtropics (Corlett and Hughes, 2015). These forests provide invaluable ecological, economic, social, and aesthetic benefits, particularly in the areas of biodiversity conservation, wood products, food and medicinal provisions, and regulation of regional and/or local hydroclimate (Bonan, 2008). Most of the original natural forest, however, has been removed by intensive agricultural and human disturbance, and has now been replaced by young secondary forests and monocultural plantations, except at high elevations (Corlett and Hughes, 2015). Various forest community types, including both pristine and human-disturbed communities, provide ideal areas to calibrate the phytolith–vegetation relationship in extratropical zones, and doing so would also help evaluate the effect of climate and humans on forest composition, structure, and evolution.

Previous phytolith reference investigations have demonstrated that phytolith types and production associated with broadleaf trees in northern (temperate) and southern (subtropical) China have no detectable differences (*cf*. Wang and Lu, 1993; Lu et al., 2006; An et al., 2015; Gao et al., 2018) despite tremendous variations in regional climate and forest type. This suggests diagnostic phytolith morphotype(s) that could directly distinguish subtropical forest from temperate forest in China is lacking. However, using morphological and statistical approaches, several recent studies have indicated that phytolith analysis should be considered a reliable tool for specifying forest communities and distinguishing grasslands from forested habitats in the northern temperate zone (Gao et al., 2018). For the subtropical evergreen forest zone, where grass is usually absent or uncommon and evergreen vegetation displays less seasonality (Corlett and Hughes, 2015), the number of phytolith-based studies related to subtropical non-grass and dicot species is quite limited. Phytolith assemblages from subtropical topsoil under different vegetation communities are still understudied, and this has hampered the application of phytolith analysis to paleoecological reconstructions in the subtropics. To overcome this limitation, analyses of topsoil phytolith assemblages and present vegetation could provide important information on representation, abundance, and characteristics of phytoliths in subtropical forests, and thus provide a fundamental reference for the interpretation of the fossil phytolith assemblages preserved in geological archives within this region.

The research reported here investigates the potential and limitations of phytolith assemblages for reconstructing vegetation and tree cover density in the subtropical zone. This initial investigation was accomplished by studying topsoil phytolith assemblages under different vegetation communities along an elevation transect in subtropical mountains of southeastern China. Referring to all available modern phytolith data, this work examines how modern vegetation communities in the monsoonal subtropical forest can be discriminated with phytolith assemblages. The resulting phytolith-based vegetation estimates are compared to satellite data to assess the potential for using phytolith assemblages as interregional and taxa-free proxies of vegetation coverage. The following questions are addressed: (1) Do phytolith assemblages reliably distinguish different forest communities within the subtropical zone? (2) What is the most important factor governing the variability in topsoil phytolith assemblages? (3) Can phytolith analysis serve as an alternative or complementary proxy to pollen? To answer the last question, the efficacy of phytolith analysis is further assessed using a modern pollen rain dataset collected from the same transect (Qiu, 1993).

## Materials and Methods

### Regional Setting

To study a vegetation community with minimum anthropogenic disturbance, the Daiyun Mountains National Nature Reserve (25°38′07″-25°43′40″N, 118°05′22″-118°20′15″E) in central Fujian province of southeastern China was selected. The national reserve was created to preserve the natural subtropical mountain forest ecosystem, which represents one of the most common forest ecosystems in southeastern China (Lin, 2003). Within the core reserve area, farming and stockbreeding are not allowed.

The Daiyun Mountains region is characterized by a maritime monsoon climate. The lowest mean monthly temperatures occur in January and range from 6.5 to 10.5°C; the highest temperatures occur in July and vary from 23.0 to 27.5°C (Lin, 2003). Mean annual temperature for the study area ranges from 15.6 to 19.5°C, while mean annual precipitation in the region extends from 1,700 to 2,000 mm. More than 85% of the annual precipitation falls from March to September each year. The yearly average relative humidity is above 80%.

The average elevation of the reserve is more than 1,200 m, with a maximum elevation of 1,856 m, which represents the second tallest mountain in Fujian Province. Because of the significant elevation differences, the natural vegetation varies from broadleaf evergreen forest (900–1,000 m), broadleaf evergreen-deciduous mixed forest (1,000–1,300 m), coniferous-broadleaf mixed forest (1,100–1,300 m), warm/temperate coniferous forest (1,300–1,500 m), to evergreen shrub-meadow (>1,500 m). The natural variation in forest communities along the elevation gradient, as well as their climatic differences, provides a unique opportunity to verify the applicability of using phytolith assemblages to differentiate forest communities in the subtropics of China.

### Sample Collection

**Figure 1:**
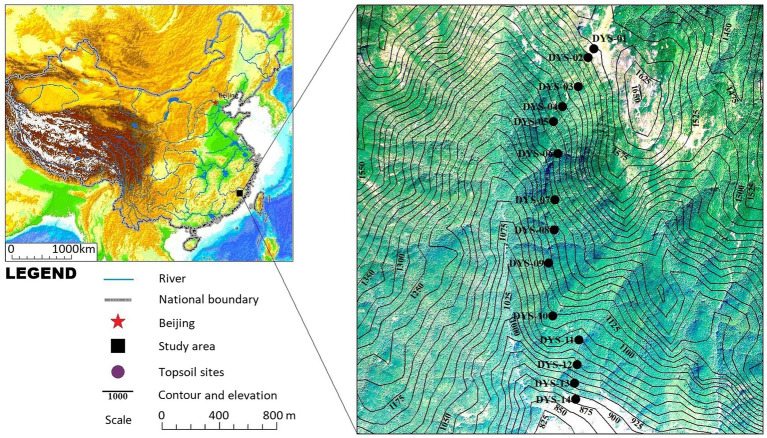
Location of the study area and topsoil sampling sites (Table 1) along the elevation gradient in the Daiyun Mountains, southeastern China.

In October 2020, 14 topsoil samples were collected from the different vegetation communities along the elevation gradient of the Daiyun Mountains (peak elevation of 1,664 m). The samples were collected from south to north on 14 contiguous plots located along a 700-m elevation transect (Figure 1). Each plot was 10 × 10 m^2^, except those for shrub-meadow samples, which spanned 2 × 2 m^2^. Each sample consisted of 3–5 subsamples (5 × 5 × 1 cm^3^) of soil or litter collected at the four corners and center of each quadrat and then mixed together. Each mixed sample represents the average of the modern phytolith grains produced by the decomposition of above-ground vegetation over a few years. The geographical coordinates, elevation, and dominant species for each quadrat are summarized in Table 1.

**Table 1:** Location, elevation, and dominant plant species for topsoil samples collected in the Daiyun Mountains.

Vegetation type	ID	Long. (°)	Lat. (°)	Elev. (m)	Quadrat size (m^2^)	Dominant plant species
Evergreen shrub-meadow	DYS-01	118.20073	25.66939	1,572	2 × 2	Shrub layer: *Pinus taiwanensis*, *Ilex crenata*, *Stranvaesia davidiana* var. *undulata*, *Eurya saxicola* Ground layer: *Miscanthus sinensis*, *Arundinella anomala*, *Deyeuxia arundinacea*, *Isachne truncata*, *Veratrum japonicum*, *Carex* spp.
	DYS-02	118.20023	25.66892	1,537	2 × 2	Shrub layer: *Pinus taiwanensis*, *Eurya saxicola*, *Vaccinium japonicum* var. *sinicum*, *I. crenata*, *S. davidiana* var. *undulata* Ground layer: *M. sinensis*, *Arundinella anomala*, *D. arundinacea*, *Isachne truncata*, *Carex* spp., *Woodwardia japonica*, *Diplopterygium glaucum*
	DYS-03	118.19924	25.66725	1,481	2 × 2	Shrub layer: *Pinus taiwanensis*, *Eurya saxicola*, *Eurya nitida*, *Rhododendron fortune*, *Rhododendron mariesii*, *Clethra cavaleriei*, *Rhododendron simsii* Ground layer: *Woodwardia japonica*, *Dicranopteris pedata*, *M. sinensis*, *Veratrum japonicum*, *Aster ageratoides*, *Carex* spp., *Lophatherum gracile*
	DYS-04	118.19797	25.66620	1,401	2 × 2	Shrub layer: *Cyclobalanopsis gracilis*, *Pinus taiwanensis*, *Rhododendron mariesii*, *Rhododendron latoucheae*, *E. nitida*, *Eurya saxicola*, *Vaccinium bracteatum* Ground layer: *Woodwardia japonica*, *Carex* spp., *Arundinella anomala*, *Dicranopteris pedata*, *M. sinensis*, *Isachne truncata*, *Lophatherum gracile*
Coniferous- broadleaf mixed forest	DYS-05	118.19719	25.66539	1,359	10 × 10	Canopy layer: *Cyclobalanopsis glauca*, *Camellia octopetala*, *Ilex rotunda*, *Michelia maudiae*, *Dendropanax dentiger*, *Pinus taiwanensis*, *Castanopsis faberi*, *Schima superba*, *Lithocarpus glaber* Shrub layer: *Lindera aggregata*, *Sarcandra glabra*, *Symplocos lancifolia*, *E. nitida*, *Rhododendron mariesii*, *Rhododendron simsii* Ground layer: *Woodwardia japonica*, *D. glaucum*, *Lophatherum gracile*
Bamboos	DYS-06	118.19712	25.66339	1,319	10 × 10	Canopy layer: *Phyllostachys edulis* Shrub layer: *P. edulis*, *Indocalamus tessellatus*, *Oligostachyum oedogonatum*, *Dendropanax dentiger* Ground layer: *D. glaucum*
Coniferous- broadleaf mixed forest	DYS-07	118.19640	25.66062	1,278	10 × 10	Canopy layer: *Castanopsis faberi*, *Pinus taiwanensis*, *Elaeocarpus japonicus*, *Cunninghamia lanceolata*, *Camellia octopetala*, *Cyclobalanopsis gracilis*, *Schima superba*, *Dendropanax dentiger* Shrub layer: *Sarcandra glabra*, *Ilex pubescens*, *Eurya weissiae*, *Vaccinium sprengelii*, *Litsea elongata* Ground layer: *Woodwardia japonica*, *Plagiogyria dunnii*
	DYS-08	118.19600	25.65879	1,229	10 × 10	Canopy layer: *Pinus taiwanensis*, *Castanopsis tibetana*, *Schima superba*, *Cyclobalanopsis glauca*, *Lithocarpus glaber*, *C. lanceolata*, *Dendropanax dentiger* Shrub layer: *Symplocos sumuntia*, *Loropetalum chinense*, *Lindera aggregata*, *V. bracteatum*, *Adinandra millettii*, *Syzygium buxifolium* Ground layer: *Woodwardia japonica*, *Plagiogyria dunnii*, *Blechnum orientale*
	DYS-09	118.19527	25.65685	1,181	10 × 10	Canopy layer: *Pinus massoniana*, *Castanopsis eyrei*, *Schima superba*, *Dendropanax dentiger*, *Toxicodendron succedaneum*, *Machilus thunbergii* Shrub layer: *Vaccinium carlesii*, *Rhododendron ovatum*, *Rhododendron simsii*, *E. nitida*, *Lindera aggregata*, *Raphiolepis indica* Ground layer: *Dicranopteris pedata*, *Woodwardia japonica*, *Pyrola calliantha*, *D. glaucum*
Broadleaf forest	DYS-10	118.19494	25.65359	1,133	10 × 10	Canopy layer: *Quercus variabilis*, *Loropetalum chinense*, *Machilus leptophylla*, *Cinnamomum subavenium*, *Rhododendron latoucheae*, *Daphniphyllum calycinum*, *Myrica rubra*, *Diospyros kaki* var. *silvestris* Shrub layer: *E. nitida*, *Symplocos sumuntia*, *Machilus grijsii*, *Camellia oleifera*, *Sarcandra glabra*, *Castanopsis carlesii*, *Dendropanax dentiger* Ground layer: *Woodwardia japonica*, *D. glaucum*
	DYS-11	118.19642	25.65186	1,056	10 × 10	Canopy layer: *P. massoniana*, *Castanopsis faberi*, *Castanopsis eyrei*, *Schima superba*, *Machilus thunbergii*, *C. lanceolata*, *P. edulis* Shrub layer: *I. tessellatus*, *Oligostachyum oedogonatum*, *Sarcandra glabra*, *Rhododendron latoucheae* Ground layer: *Woodwardia japonica*, *Dicranopteris pedata*, *D. glaucum*
	DYS-12	118.19602	25.65039	1,012	10 × 10	Canopy layer: *C. lanceolata*, *Taxus wallichiana* var. *mairei*, *Cryptomeria fortunei*, *P. edulis*, *Machilus phoenicis*, *Schima superba*, *Lithocarpus glaber*, *Cyclobalanopsis glauca* Shrub layer: *Camellia octopetala*, *I. tessellatus*, *Adinandra millettii*, *Viburnum* sp. Ground layer: *D. glaucum*, *Dicranopteris pedata*, *Woodwardia japonica*
	DYS-13	118.19564	25.64928	956	10 × 10	Canopy layer: *Castanopsis tibetana*, *Machilus phoenicis*, *Castanopsis fargesii*, *Daphniphyllum calycinum*, *Cyclobalanopsis glauca*, *Castanopsis eyrei*, *Dendropanax dentiger*, *P. edulis* Shrub layer: *Symplocos lancifolia*, *Sarcandra glabra*, *Machilus thunbergii*, *Sloanea sinensis*, *I. tessellatus*, *Oligostachyum oedogonatum* Ground layer: *Lophatherum gracile*, *Plagiogyria dunnii*, *Carex* sp.
Anthropogenic deforested shrubland	DYS-14	118.19553	25.64829	889	10 × 10	Shrub layer: *Symplocos sumuntia*, *Viburnum* sp., *P. edulis* Ground layer: *M. sinensis*, *Dicranopteris pedata*, *Lophatherum gracile*, *Arundinella anomala*, *Carex* spp., Poaceae spp.

### Phytolith Extraction

Phytoliths were extracted from the approximately 5 g subsamples of the collected topsoil using a modified version of the Piperno (1988) method (Wang and Lu, 1993). Each subsample was treated with 10% HCl to remove carbonates. Organic matter was removed using 65% HNO_3_ in a 70°C water bath. The remaining material was then subjected to gravity sedimentation separation to remove suspended clays (repeated 3–5 times) until the pH was neutralized. Phytoliths were extracted through heavy liquid (ZnBr_2_) flotation at a density of 2.35 g/cm^3^. After cleaning, each sample was rinsed in distilled water and ethanol and placed on slides in Canada balsam for counting and storage. Phytoliths larger than 10 μm were identified mainly following the classification system used by Lu et al. (2006) and Gao et al. (2018) and with reference to the classification systems of Twiss et al. (1969). Phytolith identification, photographing, and counting (usually >300 diagnostic per sample) were performed at 600x magnification using an Olympus BX53 optical microscope at Xiamen University. Phytoliths were named according to the International Code for Phytolith Nomenclature 2.0 (International Committee for Phytolith Taxonomy (ICPT), 2019).

### Climate and Satellite Data Sources and Processing

The annual and monthly climate datasets for each site (including temperature, precipitation, relative humidity, etc.) were interpolated from a network of 59 meteorological stations (Supplementary Figure S1) in Fujian Province provided by the National Climate Center, China Meteorological Administration,^1^ for the period from 1981 to 2010. Digital elevation model (DEM, 30 m) data were obtained from the NASA Shuttle Radar Topography Mission (SRTM) Global 1 arc second dataset (NASA, Jet Propulsion Laboratory (JPL), 2013). Based on climate data from, and geographical coordinates of, each station (Supplementary Figure S1), and with the aid of a geographical information system (GIS), multiple regression and residue correction methods were used to interpolate climate data for each sample site. The specific interpolation and calculation processes, as well as model performance details, are available in Liu et al. (2014).

The vegetation structure and canopy information for each sample quadrat was retrieved from long-term average (1988 to 2020) Landsat 4–8 satellite images. In this study, Enhanced Vegetation Index (EVI) and Normalized Difference Vegetation Index (NDVI) were calculated for each quadrat. All of these indices were obtained from EROS Science Processing Architecture (ESPA)^2^ on-demand interface, which offers Landsat 4–5 Thematic Mapper (TM), Landsat 7 ETM+, and Landsat 8 Operational Land Imager (OLI) original surface reflectance, metadata, and spectral indices. Quantitative tree cover data for the topsoil sample quadrats were extracted from a 2000–2012 averaged tree canopy cover dataset (Hansen et al., 2013). Although the spatial resolution of Landsat imaginary (30 × 30 m) and our sample quadrat size (10 × 10 m) differ, the Landsat imagery is of the finest resolution obtainable and is sufficient to allow an estimation of the ability to reconstruct vegetation structure characteristics from phytoliths. The interpolated climate parameters and vegetation indices for each plot appear in Table 2.

**Table 2:** Climate parameters and vegetation indices for samples collected along an elevation gradient in the Daiyun Mountains, southeastern China.

ID	Slope (°)	MAT (°C)	MAP (mm)	RH_ann_ (%)	T_Jan_ (°C)	P_Jan_ (mm)	T_Jul_ (°C)	P_Jul_ (mm)	EVI	NDVI	Tree cover (%)
DYS-01	11.09	13.04	1938.23	86.80	5.60	54.23	19.86	226.13	0.279	0.497	0.00
DYS-02	28.09	13.19	1931.23	86.64	5.72	54.15	20.04	224.60	0.296	0.617	39.96
DYS-03	24.38	13.64	1909.87	86.13	6.08	53.88	20.58	219.93	0.324	0.670	45.92
DYS-04	24.29	13.93	1896.43	85.81	6.31	53.71	20.92	216.98	0.321	0.744	83.40
DYS-05	13.83	14.10	1888.32	85.61	6.45	53.61	21.13	215.20	0.346	0.787	85.00
DYS-06	25.79	14.31	1878.46	85.37	6.62	53.47	21.38	213.13	0.306	0.796	85.00
DYS-07	24.34	14.49	1870.07	85.17	6.77	53.34	21.59	211.40	0.377	0.817	85.50
DYS-08	19.57	14.79	1856.02	84.83	7.01	53.15	21.94	208.38	0.374	0.815	89.15
DYS-09	20.36	15.04	1844.15	84.54	7.21	52.99	22.24	205.83	0.318	0.797	83.83
DYS-10	14.70	15.23	1835.40	84.33	7.37	52.85	22.46	204.06	0.440	0.808	84.02
DYS-11	18.11	15.58	1818.50	83.92	7.66	52.62	22.89	200.46	0.483	0.797	84.66
DYS-12	15.97	15.87	1805.17	83.60	7.88	52.44	23.23	197.59	0.379	0.787	85.00
DYS-13	28.42	16.12	1793.33	83.31	8.09	52.29	23.53	195.01	0.472	0.781	82.11
DYS-14	23.23	16.36	1781.75	83.04	8.29	52.14	23.82	192.50	0.445	0.672	16.10

### Statistics and Quantitative Analyses

The percentage of each phytolith type (or taxon) was calculated as a relative frequency with respect to the total phytoliths counted per sample. Diagrams of the relative frequency results were created using TILIA software (Grimm, 1992). Detrended correspondence analysis (DCA) of phytolith assemblages (not shown) demonstrated relatively short environmental gradients in the dataset, thus, redundancy analysis (RDA) was used for constrained ordination analysis. Prior to analysis, phytolith abundances for each plot were Hellinger-transformed and the environmental parameters were standardized. Ordination analysis and RDA plots were performed using the vegan 2.5–7 package (Oksanen et al., 2020) in R. Weighted averaging partial least squares (WAPLS) regression was used to quantitatively reconstruct vegetation indices for the study region with the *WAPLS()* function in the rioja package of R (Juggins, 2020). All statistical analyses were performed in R 4.0.2 (R Core team, 2020) with RStudio v 1.3.1073 (RStudio team, 2020).

## Results

### Phytolith Assemblages Under Different Vegetation Communities

**Figure 2:**
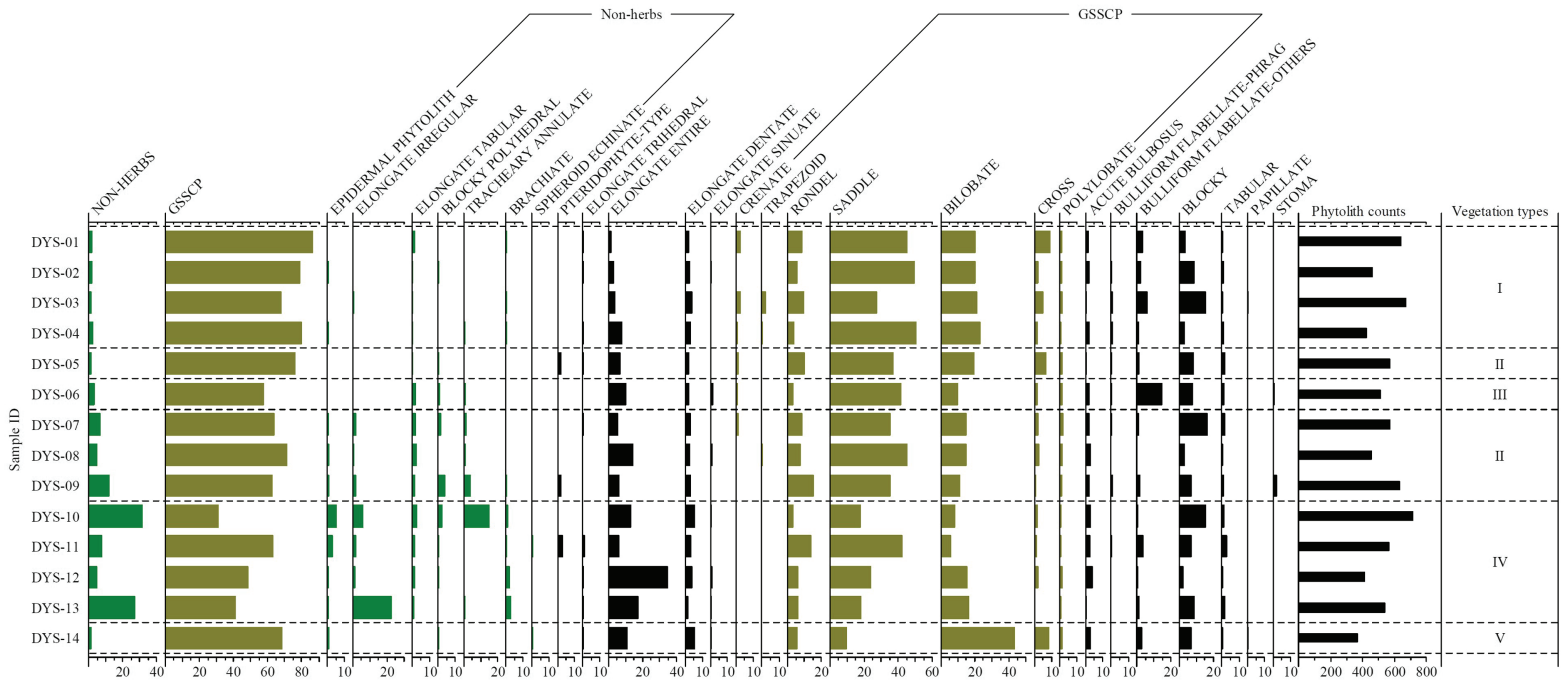
Frequency diagram of phytolith morphotypes observed in topsoil from elevation quadrats in the Daiyun Mountains, China. See Supplementary Table S1 for the full list of phytolith types counted and raw counts per sample. Group I represents evergreen shrub-meadow plots, Group II represents coniferous broadleaf mixed forest plots, Group III represents bamboo forest plot, Group IV represents broadleaf forest plots, and Group V represents anthropogenic deforested shrubland plots, respectively.

Twenty-six phytolith morphotypes were recovered from 14 surface soil samples collected from the Daiyun Mountains (Supplementary Table S1; Figure 2). The phytolith assemblage is shown in Figure 2, and a complete table of all phytolith types identified and counted is available as a supplementary table (Supplementary Table S1). Variations exist within the phytolith assemblages of each vegetation community and inter-plot. This section focuses on both different communities and inter-plot comparisons.

#### Evergreen Shrub-Meadow

**Figure 3:**
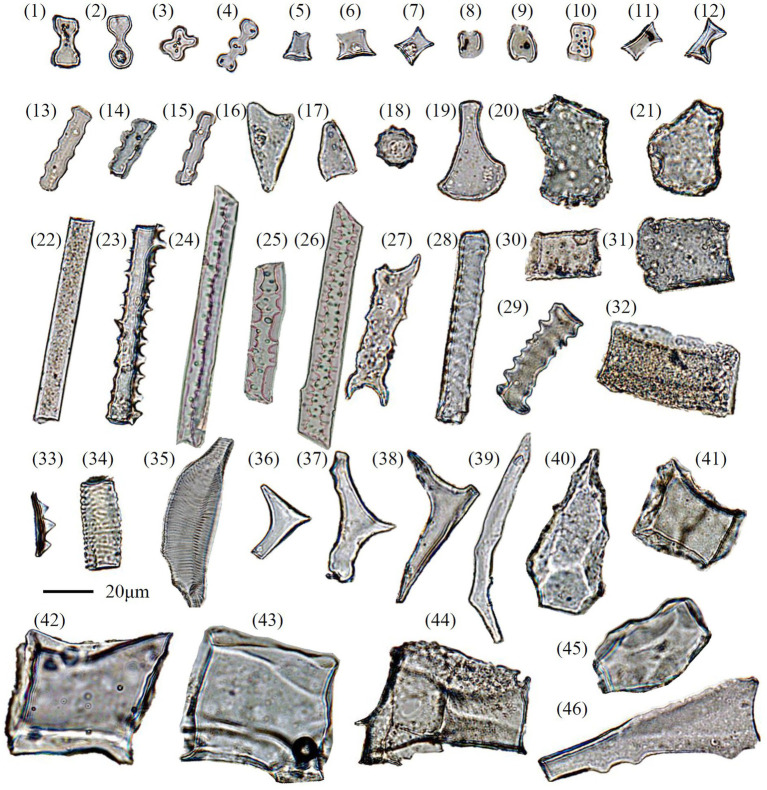
Microphotographs of selected phytolith types recovered from topsoil samples under different vegetation communities in the Daiyun Mountains, southeastern China. (1–2) Bilobate; (3) Cross; (4) Polylobate; (5–7) Rondel; (8–12) Saddle; (13–15) Crenate; (16–17) Acute bulbosus; (18) Spheroid echinate; (19–21) Bulliform flabellate; (22) Elongate entire; (23, 27, 29) Elongate dentate; (24–26) Elongate sinuate (Pteridophyte, *cf*. Lu et al., 2006); (28) Elongate tabular; (30–32) Blocky; (33) Papillate; (34–35) Tracheary annulate/helical (woody species, *cf*. Lu et al., 2006; Wen et al., 2018); (36–38) Brachiate (woody species, *cf*. Lu et al., 2006; Wen et al., 2018); (39) Elongate geniculate (woody species, *cf*. Lu et al., 2006; Wen et al., 2018); (40,45) Blocky polyhedral (woody species, *cf*. Lu et al., 2006; Wen et al., 2018); (41–44) Blocky (woody species, *cf*. Lu et al., 2006; Gao et al., 2018); (46) unclassified (woody species?).

Four plot samples were collected under evergreen shrub-meadow (DYS-01, 02, 03, 04, Table 1). The phytolith assemblages in soils of this community were dominated exclusively by grass silica shot-cell phytoliths (GSSCP), particularly Saddle, Bilobate, Rondel, and Cross, at more than 78% (Figures 2, 3). Saddle phytoliths alone constituted a significant portion of this phytolith assemblage (43% on average). The frequency of Saddle phytoliths in this community exceeded that found for this phytolith type in samples from the other communities (Figure 2), suggesting contribution from Poaceae in the evergreen shrub-meadow. Bulliform flabellate were observed in low amounts (3–6%) within these samples but its mean frequency was higher in the evergreen shrub-meadow than in any of the other communities. Arboreal phytolith frequencies were lower in this community than in any of the other natural vegetation communities analyzed (Non-herbaceous mean < 2%). The majority of Non-herbaceous phytoliths consisted of Elongate tabular, a characteristic phytolith morphotype of woody plants, especially from coniferous trees (Gao et al., 2018).

#### Coniferous-Broadleaf Mixed Forest

Coniferous-broadleaf mixed forest plots are represented by four topsoil samples (DYS-05, 07, 08, 09, Table 1). The phytolith assemblage from coniferous-broadleaf mixed forest quadrats was similar to that of evergreen shrub-meadow, particularly in terms of high GSSCP and low arboreal frequencies (Figure 2). Some differences, however, still exist. Samples from this community exhibited a slightly higher percentage of Non-herbaceous phytoliths (>6% on average), compared to less than 2% in samples from evergreen shrub-meadows. The surface soil phytolith assemblage reflected the observed vegetation inventory with the increasing contribution of woody species. The frequency of GSSCP (68%), however, was lower than from evergreen shrub-meadow. Saddle and Bilobate phytoliths occurred in lower frequencies than they did in the evergreen shrub-meadow, likely because of the decrease in Poaceae abundance and richness (see Supplementary Table S1). Another distinctive feature of the phytolith assemblage under coniferous-broadleaf mixed forest was the presence of Pteridophyte-type phytoliths, although their percentage was not high (about 1% on average).

#### Bamboo Forest

Due to its comparatively narrow environmental gradient, only one subsample (DYS-06) was collected from the bamboo forest plot. GSSCP (58%) dominated its phytolith assemblage. Here, however, the composition of GSSCP was dominated by Saddle with a notable decrease in the frequencies of Bilobate, Rondel, and Cross phytoliths compared to other plots (Figure 2). One distinctive feature of the bamboo forest phytolith assemblage is the higher percentage of Bulliform flabellate types (15%) than was found in any other studied plot (Figure 2). Morphology investigations suggested that most of the Bulliform flabellate and some of the Saddle phytoliths were produced by Bambusoideae (*cf*. Supplementary Figure S2 and Gu et al., 2016; Li R. et al., 2017). Non-herb phytoliths occurred at a moderate level, again reflecting the contribution from woody species.

#### Broadleaf Forest

Four independent quadrat samples (DYS-10, 11, 12, 13) came from the broadleaf forest plots. The majority of phytolith samples obtained from the broadleaf forest plots showed a significant increase in Elongate entire (20%) but decreases in grass silica short cell phytoliths (GSSCP mean: 46%). Non-herbaceous phytoliths, such as Blocky polyhedral, Elongate tabular, and Tracheary annulate types, were consistently present at relatively low levels in the phytolith assemblages despite broadleaf trees being the most abundant species in the vegetation inventory (Table 1). Tracheary annulate and the summed Non-herbaceous phytoliths, however, reached their highest frequencies of the bulk phytolith assemblage (about 18%), undoubtedly reflecting the dominance of woody species in this vegetation community (Figure 2). In particular, in quadrat DYS-10, Tracheary annulate phytoliths (Figure 3) accounted for 15% of the total phytoliths present and represent by far the largest occurrence of arboreal phytoliths. Understory taxa, for which data were not recorded in the botanical inventories, were relatively abundant (Figure 2) in the bulk phytolith assemblages in this community and consisted of plants from the Panicoideae (average 12%), Pooideae (average 7.5%), Chloridoideae (average 1.5%), and to a lesser extent, ferns (average 0.7%).

#### Anthropogenic Deforested Shrubland

Only a single plot (DYS-14) was sampled within the human-disturbed shrubland (deforested evergreen broadleaf forest). The phytolith assemblage for this plot displayed similarities with assemblages from the evergreen shrub-meadow but differences were also visible (Figure 2), especially in the frequencies of Saddle (10%), Bilobate (43%), and Cross (8%). The deforested shrubland had the highest frequency of Panicoideae (mean: 51%) phytoliths (Bilobate and Cross) among all sample studied (Figure 2 and Supplementary Table S1). The differences in GSSCP frequencies reflect the floristic inventory (Table 1), whereas the subfamily Panicoideae dominated the understory layer of the deforested shrubland. Saddle no longer dominated the phytolith assemblage and presented in low amounts (<10%), which is in contrast to their widespread occurrence in the natural shrub-meadow and coniferous-broadleaf mixed communities at higher elevation sites (Figure 2). A few Non-herbaceous phytoliths were identified but at very low frequencies (Figure 2). Generally, the good accordance between the phytolith assemblage and the botanical checklist suggests that phytoliths could serve as a useful indicator to identify anthropogenic deforestation.

### Relationship Between Topsoil Phytolith Assemblage and Bioclimatic Variables

#### Response of Phytolith Assemblages to the Elevation Gradient

**Figure 4:**
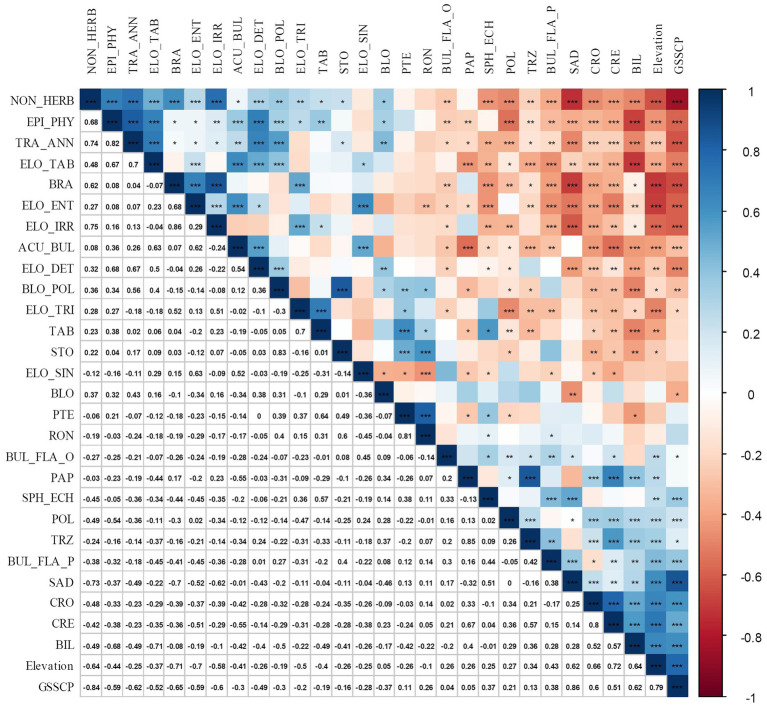
Correlation matrix made using the R package “corrplot,” between phytolith morphotype’s percentage and environmental parameters for the topsoil plots collected in the Daiyun Mountains. The legend on the right side of the diagram shows the Pearson correlation coefficients with their corresponding colors. Positive correlations are displayed in blue and negative correlations in red. *, **, and ***denote the statistical significance at *α* = 0.05, *α* = 0.01, and *α* = 0.001, respectively. Phytolith codes (abbreviations) used here are referred to International Committee for Phytolith Taxonomy (ICPT) (2019). Particularly, EPI_PHY denotes Epidermal phytolith; ELO_IRR denotes Elongate irregular; ELO_TAB denotes Elongate tabular; BRA denotes Brachiate; PTE denotes Pteridophyte-type; BUL_FLA_P denotes Bulliform flabellate that is produced by *Phragmites*; BUL_FLA_O denotes general Bulliform flabellate; TAB denotes Tabular; STO denotes silicified Stoma.

Almost all phytolith morphotypes, except the Pteridophyte-type (PTE), Elongate sinuate (ELO_SIN), Rondel (RON), and Blocky (BLO), showed significant correlation with elevation (Figure 4). The Non-herbaceous phytolith category, which is mainly composed of Blocky polyhedral, Tracheary annulate, Brachiate, Spheroid echinate, Elongate irregular, Elongate tabular, and Epidermal types, showed negative correlations with GSSCP (*r* = −0.84, *p* < 0.01, *n* = 14). With an increase in elevation, the percentages of GSSCP increased, whereas the Elongate and Non-herb phytolith decreased (Figures 2, 4).

#### Ordination Analysis Results

**Figure 5:**
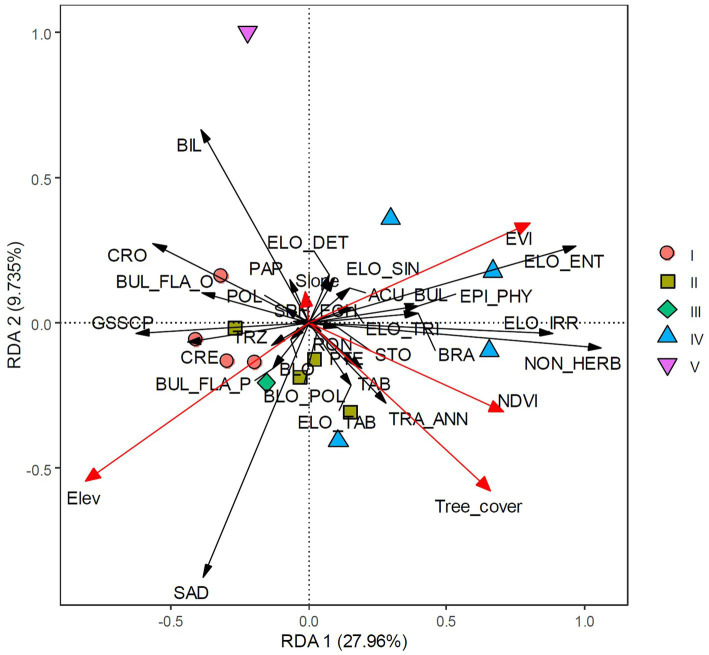
Redundancy analysis (RDA) plot of environmental parameters and phytolith taxa. Scatter points show the first two axis scores. Group I represents evergreen shrub-meadow plots, Group II represents coniferous broadleaf mixed forest plots, Group III represents bamboo forest plot, Group IV represents broadleaf forest plots, and Group V represents anthropogenic deforested shrubland plots, respectively.

Detrended correspondence analysis (DCA) suggested that a linear response model is appropriate for these data. The length of the first DCA axis was 1.238 (Table 3), indicating that the samples represent a small fraction of the environmental gradient and that species responded to the environmental change linearly. Redundancy analysis (RDA), a linear ordination method, was then applied to the phytolith data. The RDA specified that the included environmental factors explained 51.78% of the variation in the phytolith assemblages across all sites. The first and second RDA axes explained 27.96 and 9.73%, respectively, of the total variation in the phytolith assemblage (Table 3). The first RDA axis had a relatively high eigenvalue and captured 53.99% of the total explainable variation among the phytolith–bioclimate relationship. The first two axes together captured 72.70% of the explainable variations. As shown in Figure 5, almost all explanatory variables were separately distributed along the RDA axis 1. Among these variables, elevation showed a negative correlation with axis 1, whereas the vegetation indices were plotted toward the positive direction of axis 1 (Figure 5). It is evident that axis 1 represents the vegetation change induced by the elevation gradient: more negative sample loadings denote higher elevation and lower canopy coverage, whereas more positive loadings suggest lower elevation and denser canopy coverages (Figure 5).

**Table 3:** Summarized results of detrended correspondence analysis (DCA) and redundancy analysis (RDA) on the phytolith assemblages recovered from the topsoil samples collected in the Daiyun Mountains. SD denotes standard deviation units for DCA.

	Axis 1	Axis 2	Axis 3	Axis 4
DCA				
Eigenvalue	0.156	0.060	0.034	0.026
Axis lengths (SD)	1.238	0.771	0.521	0.631
RDA				
Eigenvalue	0.025	0.009	0.005	0.005
Proportion explained	0.280	0.097	0.059	0.054
Cumulative proportion	0.280	0.377	0.436	0.490

Due to the elevation and vegetation gradient, different phytolith morphotypes can be classified into two distinct groups along the RDA axis 1. Grass silica short cell phytoliths (GSSCP), such as Saddle, Bilobate, Cross, Crenate, Trapezoid, and Polylobate, were plotted separately from various Elongate types, Acute bulbosus, and Non-herbaceous phytoliths (Figure 5). Quadrats sampled from high elevation sites (evergreen shrub-meadow, coniferous-broadleaf mixed forest) were plotted along the left direction of RDA axis 1, indicating negative loadings of GSSCP along axis 1 (Figure 5). In contrast, the positive component loadings of other phytoliths were recovered from low to middle elevation plots (broadleaf forest). The second RDA component (axis 2), however, did not show such a clear trend. The single sample collected under the anthropogenically deforested shrubland, which is characterized by high Bilobate frequency, is quite different from the other sites (Figure 5).

### Quantitative Estimation of Tree Coverage

**Figure 6:**
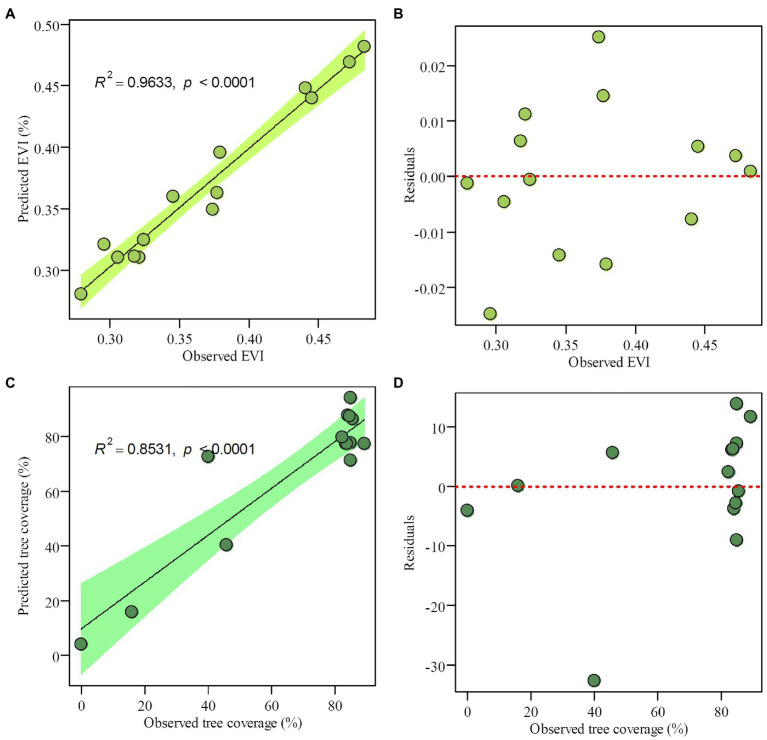
The 5-component WA-PLS inference model results for **(A)** observed versus predicted enhanced vegetation index (EVI), **(B)** observed EVI versus residual values, **(C)** observed versus inferred tree coverage (%), and **(D)** observed tree coverage versus residual values.

Employing WA-PLS regression, the phytolith-based modern canopy tree coverage estimates matched satellite data well (Hansen et al., 2013). Table 4 lists the performance of transfer functions established to estimate the enhanced vegetation index (EVI) and canopy coverage for each quadrat in the Daiyun Mountains (NDVI were not estimated because tree coverage gives similar information with NDVI, *r* = 0.9386, *p* < 0.001). The five-component WAPLS model had the most robust performance statistics (Table 4; Figure 6) for both EVI and tree coverage, with satisfactory estimates of percent variance explained and root mean squared error (RMSE) for EVI (*R*^2^ = 0.9633, RMSE = 0.0125) and tree coverage (*R*^2^ = 0.8531, RMSE = 10.9516), respectively (Figure 6). This suggests that the potential is great for canopy coverage to be quantitatively estimated in subtropical areas.

**Table 4:** Performance of WAPLS model relating to EVI and canopy tree coverage and phytolith variance.

Vegetation parameters	Model	Apparent			Cross-validation		
		RMSE	*R* ^2^	Max bias	RMSE	*R* ^2^	Max bias
EVI	WAPLS-1	0.0469	0.4822	0.0890	0.0575	0.2358	0.0030
	WAPLS-2	0.0336	0.7341	0.0312	0.0751	0.0580	0.0014
	WAPLS-3	0.0198	0.9074	0.0284	0.0854	0.0442	−0.0035
	WAPLS-4	0.0144	0.9516	0.0220	0.0825	0.0685	−0.0046
	WAPLS-5	0.0125	0.9633	0.0142	0.0853	0.0979	−0.0088
Tree coverage (%)	WAPLS-1	22.5233	0.3791	47.7525	29.1679	0.0962	59.3434
	WAPLS-2	16.9231	0.6495	38.6007	28.2266	0.1734	61.9126
	WAPLS-3	15.4154	0.7093	25.9651	33.4372	0.1342	63.3994
	WAPLS-4	13.1095	0.7896	26.4303	47.2017	0.0306	74.5377
	WAPLS-5	10.9516	0.8531	32.6224	61.2644	0.0011	81.3634

## Discussion

### Reliability and Limitations of the Survey

#### Accordance Between Field Vegetation Inventory and Phytolith Assemblages

Comparing the phytolith assemblage to the floristic checklist, generally good accordance was found between the topsoil phytolith assemblages (Figure 2) and the plant taxa documented in the specific vegetation plot inventory (Table 1). For example, in the evergreen shrub-meadow plots, various Poaceae and Cyperaceae species were encountered during the field survey, and this is faithfully reflected by the dominance of GSSCP. However, differences still exist. For instance, *Phragmites* Bulliform flabellate (<1% on average) was recovered from three of four evergreen shrub-meadow plots, but *Phragmites* species were not mentioned in the species list. Moreover, ferns were recorded in the plant checklist in the bamboo forests but diagnostic phytolith morphotypes of pteridophyte were not recognized within the surface soil phytolith assemblages. Those phytoliths may represent inheritance from past vegetation or input from a nearby quadrat transported into the study plot by aeolian processes or surface runoff (Dickau et al., 2013; Watling et al., 2016). The contribution of those extraneous phytoliths might increase the uncertainty of topsoil phytolith-based climate or vegetation estimation models, especially when dramatic differences exist between current and past vegetation. However, as reflected by the phytolith frequency diagram, the contribution of extraneous phytoliths to the bulk phytolith assemblages, was comparatively low (despite not being able to accurately estimate how many phytoliths were inherited or transported from past/nearby vegetation). Our phytolith sampling array generally reflected the alternations in natural vegetation communities: the percentages of GSSCP increased from lower elevation sites to higher elevation sites, whereas the silica long cell phytolith and Non-herb phytolith decreased (Figures 2, 4).

Differences in representativeness between arboreal and herbaceous (or grass) phytoliths, however, were also significant in our study, which is consistent with previous calibrations (Piperno, 1988; Wang and Lu, 1993; Bremond et al., 2004; Hyland et al., 2013; Crifò and Strömberg, 2021). Topsoil phytolith assemblages of all samples were dominated exclusively by GSSCP, suggesting a strong over-representativeness of GSSCP to Poaceae species (Figure 2). Even in the broadleaf forest quadrat, Non-herbaceous phytoliths, accounted for less than 18% (on average) of the bulk phytolith assemblage, whereas GSSCP represented approximately 46% of the total phytoliths (Figure 2). Since GSSCPs are produced abundantly by all parts of Poaceae, grass presence in the samples might be significantly over-represented. In contrast, as most arboreal phytoliths are produced in the leaves of the trees in subtropical evergreen forests, the under-representation of arboreal phytoliths to trees is not surprising (Piperno, 1988; Wang and Lu, 1993). Future additional calibrations are needed to address the representativeness differences between arboreal and herbaceous phytoliths, as has been done in palynology (e.g., Sun et al., 2016; Jiang et al., 2020; Fang et al., 2022). Regardless, relative abundances of arboreal and grass phytoliths varied consistently with natural vegetation changes along the elevation gradient, suggesting that phytolith assemblages might be a useful tool for quantitative vegetation reconstruction.

#### Robustness of the Phytolith-Based Canopy Coverage Transfer Function

Because of intense deforestation activities in subtropical evergreen forests, the natural (or less disturbed) vegetation is restricted to high mountain areas. In this study, only 14 plots were sampled under different vegetation communities along the relatively short elevation transect in the Daiyun Mountains of subtropical China. It is not possible, therefore, to establish a robust phytolith assemblage-based quantitative estimate of canopy coverage for the entire subtropics based on the results of this study alone. As shown in Table 4, although the five-component WA-PLS model predicted EVI and tree coverage in close approximation to the observations obtained from satellite imagery (Figure 6), leave-one-out cross-validation suggested that the five-component WA-PLS model did not have a strong coefficient of determination (*R*^2^ < 0.1) nor a low root mean square error of prediction. This might be attributable to the small size of the training dataset: when fewer samples are considered, the robustness of the transfer function is challenged.

Turner et al. (2021) postulated that the robustness of a transfer function depends largely on the sampling range and the continuity of modern reference sites rather than on the number of samples. Despite the short sampling transect in the present study, the tree coverage of the studied plots as estimated from satellite data ranged from 0 to 89% (Table 2), spanning a wide range of canopy coverage that may represent the different vegetation types. However, the training dataset included more dense plots (forest plots that have denser canopy coverage) than sparse plots (shrub-meadow plots with less dense canopy cover). In leave-one-out cross-validation, the removal of those less but important plot points might significantly increase the discontinuity of modern reference sites, consequently causing significant bias and decreasing the robustness of the prediction model (Table 4).

To validate the effect of a small training dataset, when the initial dataset was expanded by one time (each training subsample replicates once without incorporating any new data), the robustness of the predictive model is significantly improved (Supplementary Table S2). Cross-validation of the WA-PLS transfer function based on 28 samples yielded significantly increased coefficients of determination and much lower root mean square errors of prediction (Supplementary Table S2). The limited stability of the 14-sample-based canopy coverage prediction model is attributable to the small size of the modern sample array. Therefore, each plot included in our sample array is vital for establishing a reliable canopy coverage prediction model, and the set of all samples should be treated as a whole.

Despite the above caveats, canopy coverage in subtropical forests can be estimated effectively by topsoil phytolith assemblages. In the future, incorporating more data points from the study area might improve the transfer function. The fundamental contributions of this study, however, should remain intact: phytolith assemblages from subtropical forest topsoil are strongly connected to the openness of the landscape, and the percentage of GSSCP decreases with increasing density of canopy cover.

### Phytoliths as a Promising Tool for Canopy Coverage Reconstruction: Inter-Proxy Comparisons and Ecological Interpretations

#### Comparisons Between Phytolith and Pollen Assemblages Along the Elevation Transect

Comparisons between topsoil pollen and phytolith assemblages suggest that phytolith assemblages faithfully reflect the vegetation composition as well as pollen does. In forest landscapes, pollen has been considered more powerful than phytoliths because pollen bears finer taxonomic resolution to different plant species than phytoliths do (phytoliths can distinguish only grass-related landscapes). However, results of the present study suggest that, despite the dominance of the non-herbaceous Fagaceae (oaks and other genera) in this subtropical forest (Table 1), phytolith assemblages display vertical distribution patterns that are similar to topsoil pollen assemblages.

**Figure 7:**
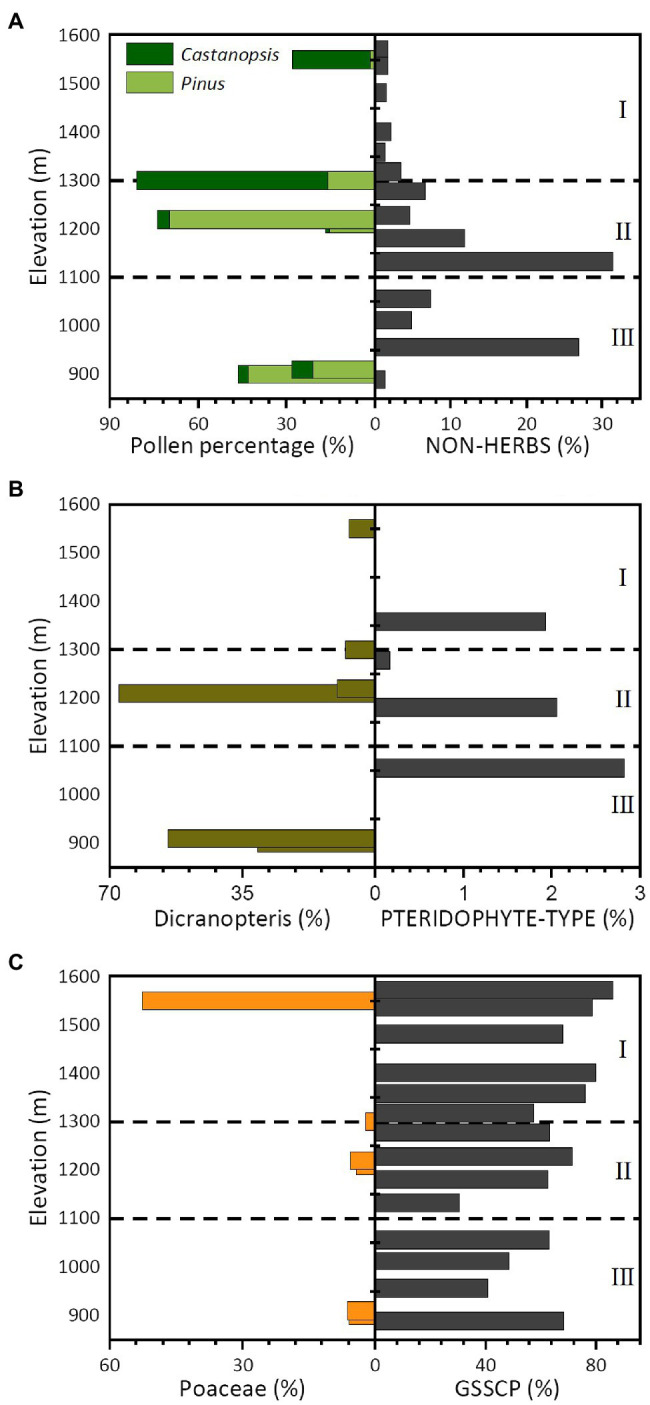
Bar chart showing the selected pollen (Qiu, 1993) and phytolith assemblages along the sampled elevation gradient in the Daiyun Mountains. **(A)** Major arboreal pollen versus Non-herb phytoliths; **(B)** *Dicranopteris* versus Pteridophyte phytoliths; **(C)** Poaceae pollen versus GSSCP.

Modern pollen assemblages collected along the elevation transect in the Daiyun Mountains were dominated by *Pinus*, *Dicranopteris*, *Castanopsis*, and Poaceae types, which account for more than 80% of the total pollen assemblage (Qiu, 1993). *Pinus* has been more frequently recorded in samples from the coniferous-broadleaf mixed and broadleaf forests but was almost absent in the shrub-meadow zone. *Castanopsis*, which was quite abundant in field plot surveys, presented low percentages below the elevation of 1,300 m but increased significantly above 1,300 m (Figure 7A). Interestingly, arboreal pollen reached its highest frequency in the coniferous-broadleaf mixed forest but was much lower in shrub-meadow (Figure 7A). Arboreal phytoliths, in contrast, had higher frequencies in coniferous-broadleaf mixed and broadleaf forests (Figure 7A). Poaceae pollen presented higher frequencies in high shrub-meadow zones, whereas *Dicranopteris* was frequently recorded in the coniferous-broadleaf mixed and broadleaf forests (Figures 7B,C). Our phytolith data agree with the pollen records: GSSCP gradually increased with elevation and Pteridophyte phytoliths were occasionally recorded in forest communities (Figures 7B,C).

Despite the general agreement, the relative abundance of each taxon shows dramatic differences between phytolith and pollen records. Arboreal pollen in coniferous-broadleaf mixed forest, for example, accounted for more than 60% of all pollen counted (Figure 7A, Qiu, 1993), but arboreal phytoliths represented no more than 6% of the phytolith assemblage. Another significant difference between pollen and phytolith records lies in their different representativeness to ferns. *Dicranopteris* spores represented more than 30% on average of the total pollen and spore assemblages in forest plots, whereas Pteridophyte phytoliths (Figure 7B) accounted for less than 1% of the phytolith assemblage. Phytoliths, moreover, may have significantly over-represented the presence of Poaceae (with GSSCP accounting for more than 65%), but Poaceae pollen comprised no more than 5% of the pollen assemblage under forest plots (Figure 7C).

Representativeness differences between pollen and phytoliths to different plant taxa agree with previous calibrations in the same or adjacent bioclimatic zones (e.g., Sun et al., 2016; Jiang et al., 2020; Fang et al., 2022). Pollen-vegetation relationship calibrations have found that R values of the *Pinus*, *Castanopsis*, and *Dicranopteris* pollen (or spores) are greater than 1 (R is the representativeness of a given pollen type, *cf*. Davis, 1963), indicating that those pollen and spore types may significantly over-represent their occurrence in subtropical forests (e.g., Sun et al., 2016; Jiang et al., 2020; Fang et al., 2022). This explains why the percentage of *Pinus* pollen was greater than 40% in broadleaf forest plots despite the almost total absence of *Pinus* species in those communities (Table 1 and Figure 7A). Strong over-representativeness was also found in *Dicranopteris* species, which usually occur in the ground layer of broadleaf or coniferous-broadleaf mixed forest types since they can survive better than other herbs under shady, humid habitats. Their frequency in the vegetation survey, however, was much lower than recorded *Dicranopteris* spores would suggest (approximately 60%). Poaceae pollen is moderately representative of Poaceae (with R value close to 1, *cf*. Sun et al., 2016; Jiang et al., 2020), and this explains why Poaceae pollen is a relatively good indicator of vegetation composition.

Phytoliths have the same problem with respect to representativeness differences. For instance, arboreal species might be significantly under-represented by arboreal phytoliths (e.g., Piperno, 1988; Dickau et al., 2013; Watling et al., 2016) because most arboreal phytoliths are produced in the leaves of the tree. Grasses, in contrast, are strong phytolith producers (*cf*. Piperno, 1988; Dickau et al., 2013; Watling et al., 2016). As a consequence, GSSCP that is diagnostic of Poaceae usually dominates the phytolith assemblages even where they are rare in the vegetation inventory.

Significant differences in dispersal, transport, and taphonomic processes are some other remaining factors accounting for the discrepancies between pollen and phytolith records. In mountainous areas, convectional uplift can cause significant upslope transportation of pollen (Qiu, 1993). *Castanopsis* species, for example, dominated the broadleaf forest understory (vegetation inventory) but were almost absent in the pollen assemblage. In shrub-meadow and coniferous-broadleaf mixed forest quadrats, however, *Castanopsis* dominated the pollen assemblage in topsoil (Qiu, 1993). The mismatch between the vegetation inventory and the topsoil pollen assemblage might be linked to the vertical transport of pollen (Qiu, 1993), causing additional complexities in interpreting the topsoil pollen assemblage accurately. Besides vertical transportation, topsoil pollen might be subject to selective preservation; thin-walled taxa may be destroyed during taphonomic processes resulting in lower percentages, while pollen taxa with thicker walls may survive and therefore accumulate in a comparably higher percentage (Mander and Punyasena, 2018; Chevalier et al., 2020; Fang et al., 2022). As a result, changes in the relative abundance of pollen assemblages may reflect taphonomic processes, rather than ecological factors (*cf*. Mander and Punyasena, 2018; Chevalier et al., 2020; Fang et al., 2022). For phytoliths, the aeolian transportation and selective preservation that is quite common for pollen, have less impact on soil phytolith assemblages. Due to their biogenic silica structure, phytoliths are less sensitive to aerial transportation and can be better preserved in the soil than pollen. Phytoliths faithfully recorded the above-ground vegetation and have finer taxonomic resolution for subfamilies of Poaceae. Despite the strong under-representation of arboreal species, the topsoil phytolith data in this study agree with pollen records (Qiu, 1993), reflecting the gradual canopy coverage change along the elevation gradient, and are less likely to be influenced by post-deposition transportation and taphonomic alterations.

#### Tree Coverage Governs the Phytolith Assemblage Change

Availability of sunlight to the ground layer has been shown to be the primary factor shaping the composition of and variation in herbaceous communities in time and space (e.g., Neufeld and Young, 2003; Niinemets, 2010; Plue et al., 2013; Mestre et al., 2017). The effective amount of radiation reaching the forest floor is determined by forest structural characteristics and tree species (e.g., Plue et al., 2013; Mestre et al., 2017). In evergreen broadleaf forests (lower elevation plots in this study), trees transmitted only a small proportion of sunlight to the forest floor, which depressed the grass richness and diversity but actively encouraged growth of ferns and sedges (strong shade-casting species). In other words, understory plants in dense evergreen broadleaf forests are adapted to low light availability in all seasons (Plue et al., 2013; Mestre et al., 2017). In the coniferous-broadleaf forests, however, the canopy of *Pinus taiwanensis* and broadleaf tree species is less dense and their leaves are substantially thinner than those of evergreen forest species. As a result, more solar radiation reaches the understory of this high elevation forest. In addition, because the coniferous trees in this area and deciduous trees drop their leaves in autumn, there exists an additional increase in light availability for the understory (Plue et al., 2013; Mestre et al., 2017). Previous field vegetation inventories in the Daiyun Mountains (Supplementary Figure S3; Dai, 2009; Zheng et al., 2016) have also shown that the composition and biodiversity of the understory herbaceous communities are mainly controlled by the sunlight reaching to the ground layer: shade-tolerant Pteridophyta and Cyperaceae dominate the ground layer in most forest quadrats and their dominance generally decrease with increasing elevation (Supplementary Figure S3; Dai, 2009; Zheng et al., 2016). Sunlight-demanding and dryness-tolerant Poaceae and Compositae start to dominate the ground layer in the shrub-meadow located at the top of the mountain (Supplementary Figure S3; Dai, 2009; Zheng et al., 2016).

**Figure 8:**
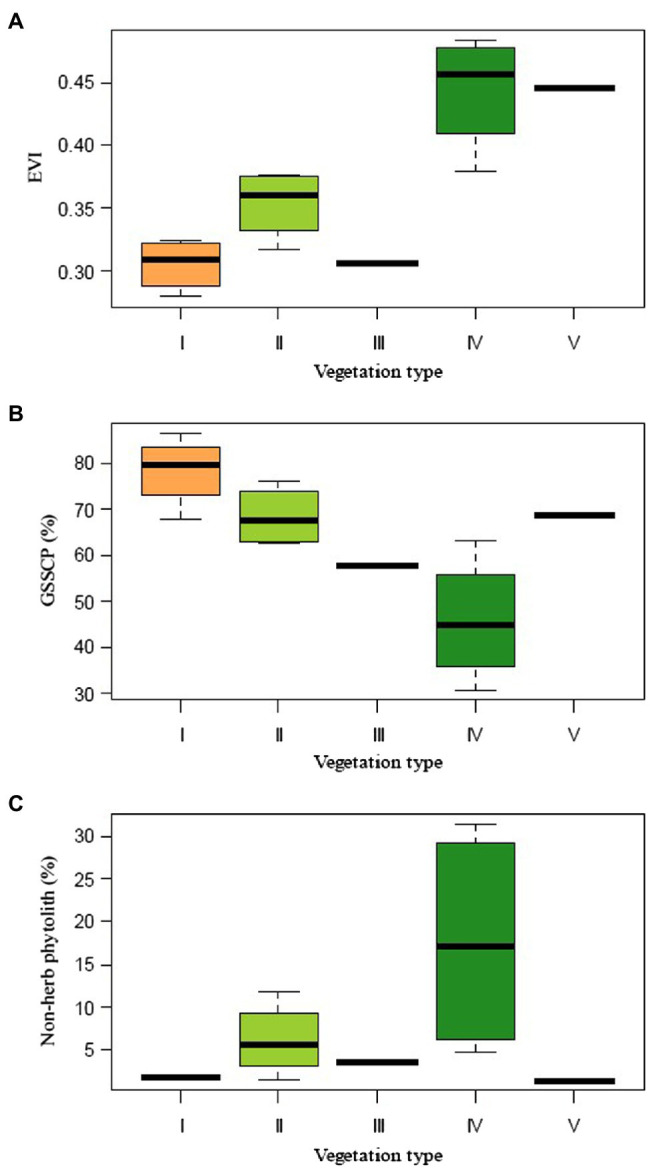
Summary of different vegetation index and phytolith percentages for different plant communities collected along the elevation gradient in the Daiyun Mountains. **(A)** Enhanced vegetation index (EVI); **(B)** Grass silica short cell phytolith (GSSCP); and **(C)** Non-herb phytolith percentages.

The present study shows that the topsoil phytolith assemblages, although mostly produced by herbs, faithfully reflect the influence of canopy structure on understory structure and composition. Overall GSSCP richness is related to the forest type, following the order shrub-meadow > mixed forest > evergreen forest (Figure 8). This is in good accordance with field floristic studies, which have underscored that the diversity of the herbaceous layer is greater in the shrub-meadow community than in the mixed and evergreen forest. While coniferous-broadleaf forests are similar to evergreen broadleaf forests, the former may facilitate diversity and/or richness of the herbaceous layer by providing a heterogeneous environment allowing the co-existence of common shade-tolerant species along with light-demanding grasses (Plue et al., 2013; Zheng et al., 2016; Mestre et al., 2017). Canopy coverage control of sunlight remains the most likely driver behind the observed changes in phytolith assemblages. The prolonged period of quantitatively low (high canopy density) or even qualitatively decreasing light availability is likely to be the factor directly responsible for this marked decline in abundance, diversity, and/or frequency of grass short-cell phytoliths.

### Implications for Paleoclimate and Paleovegetation Reconstruction

Using modern topsoil samples collected under different bioclimatic conditions, it has been proposed that variations in phytolith assemblages reflect changes in climate variables and therefore can be used for vegetation and climate reconstruction (e.g., Lu et al., 2006, 2007; An et al., 2015; Biswas et al., 2021). One fundamental assumption in that notion is that all species have climatic optima for efficient growth (e.g., Yu et al., 2004; Zheng et al., 2008). These optima, as well as extreme limits, can be reconstructed using pollen or phytolith evidence from plants (*cf*. Yu et al., 2004; Lu et al., 2006, 2007; Zheng et al., 2008).

Calibrating phytolith assemblages as a potential quantitative proxy for temperature or precipitation is more challenging than doing so with pollen mainly because of the multiplicity and redundancy characteristics intrinsic to the production of phytoliths (*cf*. Piperno, 1988; Barboni and Bremond, 2009). In addition, Poaceae, the most important phytolith producer, comprises about 10,000 species in approximately 700 genera that are distributed globally (e.g., Finot et al., 2011). The wide ecological amplitude of Poaceae, as well as the comparably narrow sampling gradient in the present investigation, means that a reliable transfer function which could be used to quantitatively estimate a climate variable for the subtropical area might be challenging. Because this investigation was conducted within a limited area of a 700-meter transect in the subtropical zone, the climatic gradients are limited: the mean annual temperature for each site ranges from 13.04°C to 16.36°C, whereas the mean annual precipitation varies from 1781.75 mm to 1938.23 mm (Table 2). However, the tree coverage gradient for our sampling array is relatively larger: the canopy coverage ranges from 0 to 89.15% (Table 2). Therefore, instead of trying to perform regression between climate parameters and phytolith assemblages, we here established the phytolith-based model to estimate the canopy coverage, since the most direct and intrinsic reason for changes in phytolith assemblages is change in vegetation (Dunn et al., 2015).

Due to the local depositional characteristics, phytoliths might perform better than other botanical indicators in quantitative canopy coverage reconstructions (*cf*. Dunn et al., 2015; Birks et al., 2016). They may help scientists better understand the ramifications that changes in global to local scale climate have on vegetation dynamics and on climate-vegetation feedbacks (e.g., Dunn et al., 2015). Caution, however, should be used when interpreting fossil (modern) phytolith assemblages because (1) Poaceae are the biggest producers of phytoliths, but also a large family whose species can adapt to different bioclimate conditions and are therefore distributed globally (e.g., Finot et al., 2011); (2) The complexity and redundancy of phytoliths may interfere with the correct identification of parent plants, especially for grass (*cf*. Piperno, 1988; Barboni and Bremond, 2009); (3) Differences in the representativeness of arboreal and herbaceous (particularly grass) phytoliths may lead to over-estimation of grass and underestimation of trees; (4) Anthropogenic deforestation could increase the canopy openness and consequently increase the frequencies of GSSCP (Figure 2) in a process that is independent of climate (Qiu, 1993). Caution must be exercised when using phytolith assemblages for quantitative paleoclimatic reconstruction lest it be interpreted incorrectly.

This study, along with others, contributes to the calibration of the climatic and vegetation significance of topsoil phytoliths (e.g., Lu et al., 2006, 2007; An et al., 2015; Biswas et al., 2021). The present study, however, is also important in linking phytolith assemblages to canopy coverage in the subtropical forest of China, with the frequency of grass short-cell phytoliths depending more on canopy coverage than climate factors directly. This explains topsoil phytolith investigations from the temperate forest-steppe ecotone where GSSCP account for 50 ~ 70% of the total phytolith assemblage (e.g., Li D. et al., 2017, 2018; Gao et al., 2018). Our analysis also supports the notion that human-induced deforestation may cause an increase in GSSCP that is independent of any climate variable. Future studies should collect data from different settings to understand how local climate and vegetation may change the phytolith–vegetation (climate) regressions in order to promote the application of phytolith analysis for paleoclimate and paleoelevation reconstructions in the subtropics.

## Concluding Remarks

This study provided fundamental information pertaining to the interpretation of fossil phytolith assemblages recovered from various sedimentary archives in the subtropics of monsoonal China. Phytoliths from 14 modern soil samples under different vegetation covers were examined and described. Analysis of topsoil samples along the elevation transect in the Daiyun Mountains revealed significant differences in phytolith morphotype composition that generally reflected species composition of the standing vegetation, especially the understory composition of different forests. While individual species cannot be detected, phytolith analysis could identify broadleaf forest, coniferous-broadleaf forest, and shrub-meadows: non-grass phytoliths appear to be the most distinctive morphotypes, but their relative abundance in vegetation might be significantly under-represented in phytolith assemblages. Grass phytoliths were the most abundant phytolith morphotypes but may over-represent the Poaceae species.

Study of phytolith–environmental relationships showed that certain morphotypes occurred preferentially in certain vegetation (climates) and at certain elevations. For example, GSSCP tended to occur in higher proportion in open habitats at higher elevations, while non-grass morphotypes occurred under mixed and broadleaf forests at lower elevations. Pteridophyta were generally more abundant in samples from moist habitats under dense broadleaf forests. The structure and openness of the canopy cover most likely modulates variations in phytolith assemblages along the evaluation gradient. We therefore established a WAPLS transfer function for future quantitative estimates of tree coverage for the study area. Our study implies that future quantitative and qualitative analyses of biogenic silica in subtropical mountainous soils (sediments) may provide a record of past fluctuations in elevation of woody versus herbaceous vegetation during the late Quaternary, triggered by variations of climate and/or by the impact of human activities. Sampling a wider range of sites across longer environmental gradients than used in this study could further improve and/or validate our understanding of the phytolith–vegetation–climate relationships.

## Data Availability Statement

The original contributions presented in the study are included in the article/Supplementary Material, further inquiries can be directed to the corresponding authors.

## Author Contributions

NL and FY designed the research. NL organized the field work with GT and SL. NL performed the research. NL, FY, and ZH analyzed the data. NL, DS, and FY wrote the paper. All authors contributed to the article and approved the submitted version.

## Funding

This work was financially supported by the China Postdoctoral Science Foundation (Grant No. 2021M691862 to NL); the National Natural Science Foundation of China (Grant No. 42076207 to FY); and the Fundamental Research Funds for the Central Universities (Grant No. 0050-ZK1116 to FY). NL received funding from the Outstanding Postdoctoral Scholarship of the State Key Laboratory of Marine Environmental Science at Xiamen University, China.

## Conflict of Interest

The authors declare that the research was conducted in the absence of any commercial or financial relationships that could be construed as a potential conflict of interest.

## Publisher’s Note

All claims expressed in this article are solely those of the authors and do not necessarily represent those of their affiliated organizations, or those of the publisher, the editors and the reviewers. Any product that may be evaluated in this article, or claim that may be made by its manufacturer, is not guaranteed or endorsed by the publisher.

## Footnotes

^1^ http://data.cma.cn

^2^ https://espa.cr.usgs.gov/
