# Supplementary material for: Phytolith Assemblages as a Promising Tool for Quantitative Canopy Coverage Reconstruction in Subtropical Forests, China
Source: Front Plant Sci. 2022 Jun 20;13:912627. doi: 10.3389/fpls.2022.912627 (PMC9251495; doi:10.3389/fpls.2022.912627)
Supplement: Supplementary file 1 [file Data_Sheet_1.PDF]

Supplementary materials

Introduction

The supplementary materials include three figures and two tables.

The data that support this study were summarized in an independent sheet in the supplementary Excel file. The supplementary raw phytolith counting was separately uploaded to this document.

Supplementary figures

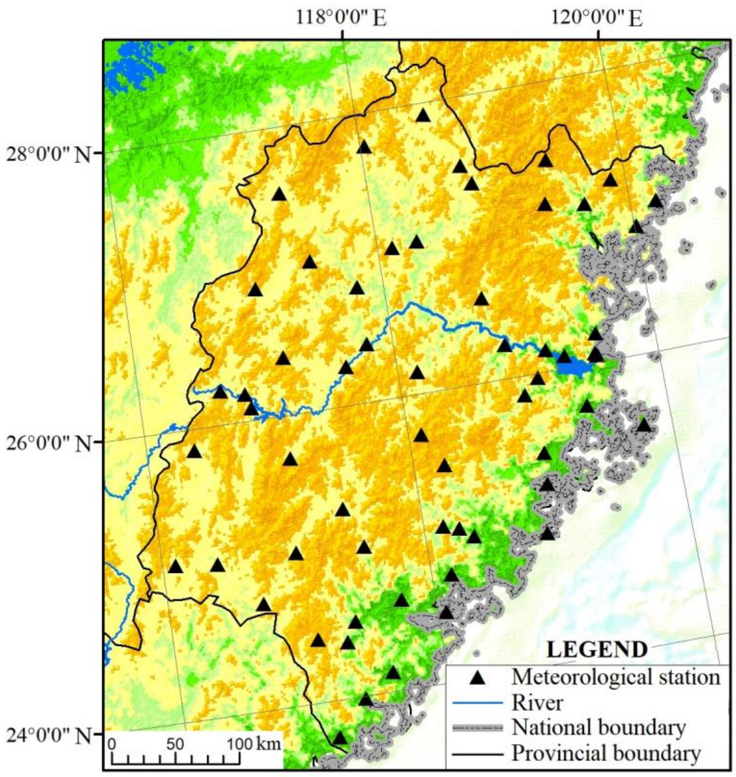

Figure. S1. Locations of 59 meteorological stations in Fujian province, China.

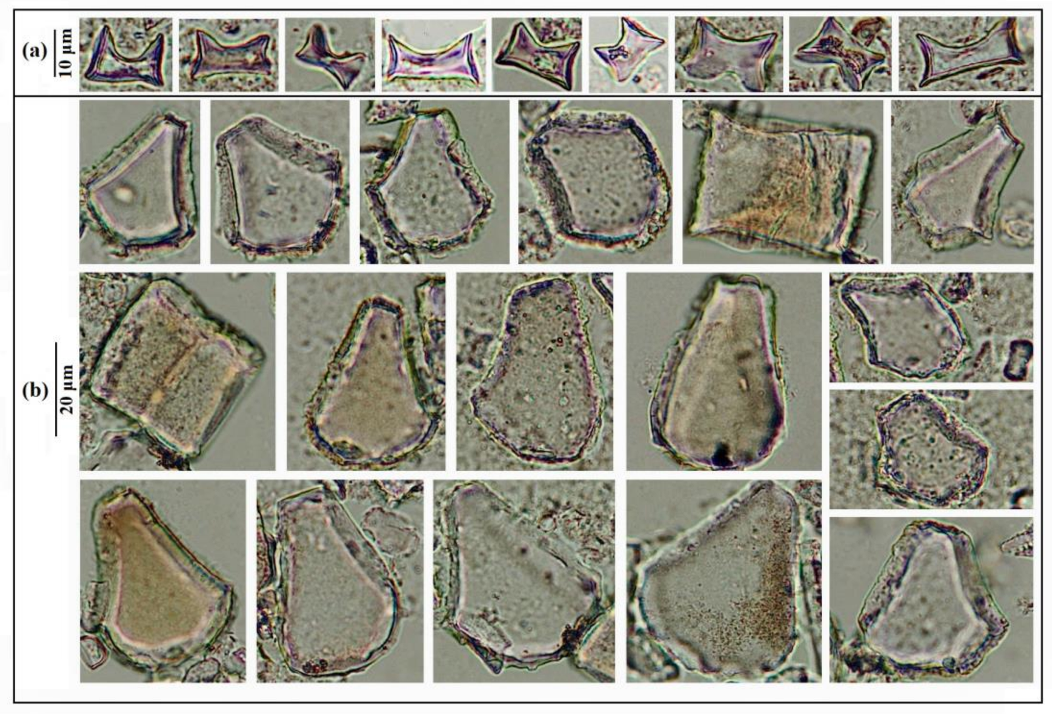

Figure. S2. Microphotographs of phytoliths extracted from topsoil sample DYS-06 that might be attributed to Bamboo (Bambusoideae). (a) RONDEL; (b) BULLIFORM FLABELLATE (cf. Gu et al., 2016; Li et al., 2017).

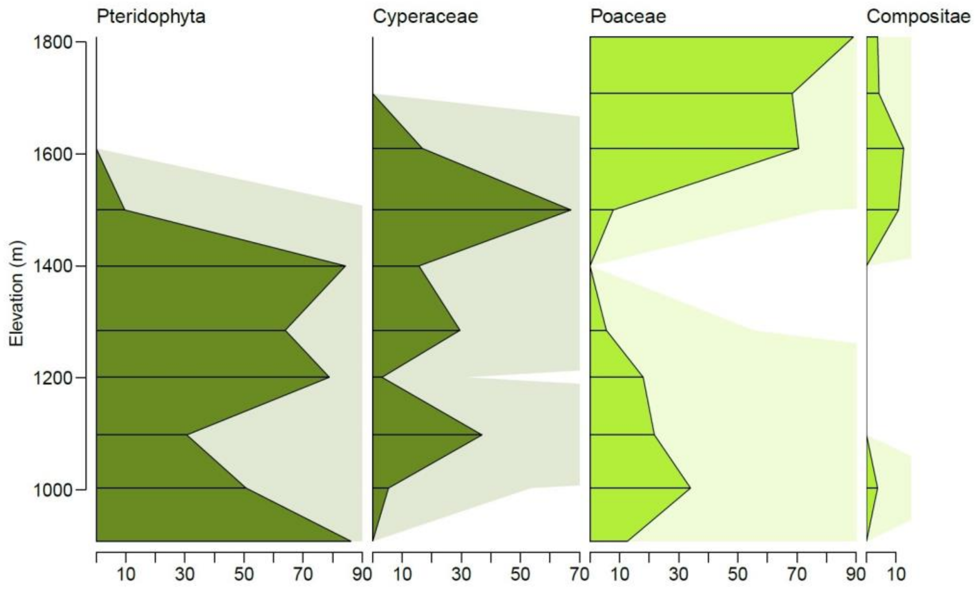

Figure. S3. Composition of the herbaceous layer from the plots investigated along the elevation gradient of the Daiyun Mountains, southeastern China (data from Dai, 2009).

Supplementary tables

Table S1. Raw phytolith data of topsoil samples from the elevation gradient in the Daiyun Mountains, China.
(Table S1 is uploaded in a separate file to this document)

Table S2. Performance of 28-subsample-based transfer function, using WAPLS model relating to EVI, and canopy tree coverage and phytolith variance.

Vegetation parameters	Model	Apparent			Cross validation		
		RMSE	R^2	Max Bias	RMSE	R^2	Max Bias
EVI	WAPLS-1	0.0469	0.4822	0.0890	0.0516	0.3793	0.1006
	WAPLS-2	0.0336	0.7341	0.0312	0.0482	0.4733	0.0572
	WAPLS-3	0.0198	0.9074	0.0284	0.0365	0.6885	0.0442
	WAPLS-4	0.0144	0.9516	0.0220	0.0211	0.8959	0.0281
	WAPLS-5	0.0125	0.9633	0.0142	0.0178	0.9259	0.0216
Tree coverage (%)	WAPLS-1	22.5233	0.3791	47.7525	25.3970	0.2350	52.8768
	WAPLS-2	16.9231	0.6495	38.6007	20.3452	0.4964	48.8609
	WAPLS-3	15.4154	0.7093	25.9651	21.0004	0.4925	42.2048
	WAPLS-4	13.1095	0.7896	26.4303	20.5953	0.5377	33.5263
	WAPLS-5	10.9516	0.8531	32.6224	17.9808	0.6150	41.1821
